# Genomic and Transcriptomic Analysis of a Patient with Early-Onset Colorectal Cancer and Therapy-Induced Focal Nodular Hyperplasia: A Case Report

**DOI:** 10.3390/jpm14060639

**Published:** 2024-06-15

**Authors:** Mary O’Reilly, Aleksandar Krstic, Luis F. Iglesias-Martinez, Éanna J. Ryan, Bruce Moran, Des Winter, Kieran Sheahan, Ray McDermott, Walter Kolch

**Affiliations:** 1 Systems Biology Ireland, School of Medicine, University College Dublin, D04 V1W8 Dublin, Ireland; aleksandar.krstic@ucd.ie (A.K.); luis.iglesiasmartinez@ucd.ie (L.F.I.-M.); walter.kolch@ucd.ie (W.K.); 2 St. Vincent’s University Hospital, University College Dublin, D04 V1W8 Dublin, Ireland; eannaryan@rcsi.ie (É.J.R.); brucemoran@svhg.ie (B.M.); des.winter@gmail.com (D.W.); ksheahan@svhg.ie (K.S.); ray.mcdermott@tuh.ie (R.M.); 3 Conway Institute, University College Dublin, D04 V1W8 Dublin, Ireland

**Keywords:** early-onset colorectal cancer (EOCRC), colorectal cancer (CRC), focal nodular hyperplasia, RAS mutated cancers, WRN (Werner Syndrome RecQ-like Helicase)

## Abstract

Early-onset colorectal cancer (EOCRC), defined as colorectal cancer in individuals under 50 years of age, has shown an alarming increase in incidence worldwide. We report a case of a twenty-four-year-old female with a strong family history of colorectal cancer (CRC) but without an identified underlying genetic predisposition syndrome. Two years after primary surgery and adjuvant chemotherapy, the patient developed new liver lesions. Extensive diagnostic imaging was conducted to investigate suspected liver metastases, ultimately leading to a diagnosis of focal nodular hyperplasia. The young age of the patient has prompted comprehensive genomic and transcriptomic profiling in order to identify potential oncogenic drivers and inform further clinical management of the patient. Besides a number of oncogenic mutations identified in the patient’s tumour sample, including KRAS G12D, TP53 R248W and TTN L28470V, we have also identified a homozygous deletion of 24.5 MB on chromosome 8. A multivariate Cox regression analysis of this patient’s mutation profile conferred a favourable prognosis when compared with the TCGA COADREAD database. Notably, the identified deletion on chromosome 8 includes the WRN gene, which could contribute to the patient’s overall positive response to chemotherapy. The complex clinical presentation, including the need for emergency surgery, early age at diagnosis, strong family history, and unexpected findings on surveillance imaging, necessitated a multidisciplinary approach involving medical, radiation, and surgical oncologists, along with psychological support and reproductive medicine specialists. Molecular profiling of the tumour strongly indicates that patients with complex mutational profile and rare genomic rearrangements require a prolonged surveillance and personalised informed interventions.

## 1. Introduction

Early-onset colorectal cancer (EOCRC) is defined as colorectal cancer in those under 50 years of age. EOCRC is becoming increasingly prevalent with one in ten diagnoses of colorectal cancer given to someone under the age of 50 [1,2]. Many hereditary predisposition cancer syndromes increase the risk of developing colorectal cancer (CRC) [3]. However, many patients with a family history of CRC do not have an identified genetic syndrome but are known to be at an increased risk of developing CRC. This case report examines the journey from diagnosis through to surveillance of EOCRC and the associated diagnostic and therapeutic challenges.

## 2. Case Presentation

### 2.1. Diagnosis and Treatment

This is the case of a twenty-four-year-old female who presented to the emergency department of our tertiary care centre in October 2020 with an acute onset of bloody diarrhoea on a background of a one-year history of intermittent abdominal pain, weight loss and altered bowel habit. The patient had no previous medical history and had an Eastern Cooperative Oncology Group Performance Score (ECOG) of 1 [4]. The patient was a non-smoker, did not consume alcohol and did not take any regular medications nor have any allergies to any medications. No previous pregnancies were documented. Family history revealed their father had been diagnosed with EOCRC at 49 years of age and died two years after diagnosis of disease progression and their mother had been diagnosed with breast cancer at 45 years of age and died a year after diagnosis. 

During the initial admission, a colonoscopy revealed a large sigmoid lesion to be the cause of the symptoms. Histopathological examination of the lesion biopsy demonstrated a mismatch-repair proficient, poorly differentiated adenocarcinoma. Computed Tomography of Thorax Abdomen and Pelvis (CT TAP) showed circumferential thickening of the mid-descending colon, measuring 9.2 cm × 8.4 cm, and out-ruled metastatic disease. The patient’s case was discussed at the colorectal multidisciplinary team meeting, and a decision was made to proceed with primary surgery. A laparoscopic extended left hemicolectomy revealed a 12 cm poorly differentiated sigmoid tumour with perforation. No tumour budding and no lymphovascular invasion were observed. Histopathological examination of 23 excised lymph nodes excluded metastatic disease. The patient was staged as pT4N0 in accordance with the latest edition of the American Joint Committee on Cancer (AJCC) TNM system, AJCC 8th Edition: Colorectal Cancer [5]. The patient completed 11 cycles of adjuvant modified fortnightly FOLFOX-6 regimen (folinic acid 400 mg/m^2^, fluorouracil bolus 400 mg/m^2^, fluorouracil continuous infusion over 46 h 2400 mg/m^2^ and oxaliplatin 85 mg/m^2^); oxaliplatin was omitted for the eleventh and final cycle as the patient had developed Grade 3 peripheral neuropathy, as defined by the Common Terminology Criteria for Adverse Events version 5 (CTCAE) [6]. Monthly goserelin (3.6 mg) was administered in conjunction with the chemotherapy to preserve ovarian function. Comprehensive genomic and transcriptomic profiling was performed on the tumour from the resected specimen; the results are discussed below. 

### 2.2. Surveillance

**Figure 1:**
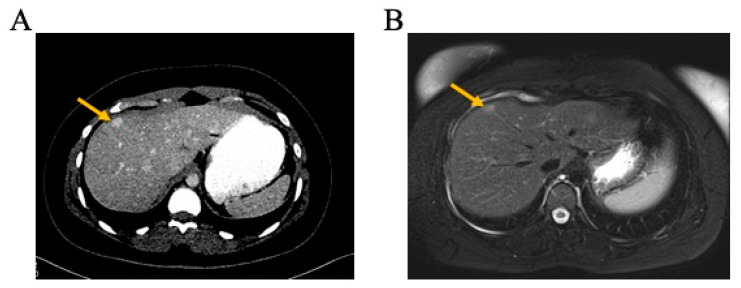
Radiological imaging of focal nodular hyperplasia. (**A**) Enhanced axial computed tomography (CT) imaging showing 2 cm liver lesion (arrow). (**B**) Same lesion as observed in image A on axial magnetic resonance imaging (MRI) (arrow).

Two years after completion of adjuvant therapy, a routine surveillance CT TAP showed two new enhancing lesions in the liver (Figure 1A). Measurement of carcinoembryonic antigen (CEA) was within the normal range at 1.8 ng/mL. Further characterisation of these lesions using contrast Magnetic Resonance Imaging (MRI) with primovist revealed four lesions in both lobes of the liver (Figure 1B). The findings were atypical for CRC metastasis, and the PET CT did not demonstrate metabolic uptake in the liver, leading to a diagnosis of focal nodular hyperplasia (FNH).

### 2.3. Genomic Profiling and Prognostic Evaluation

Due to the patient’s family history and the unusually young age at diagnosis of CRC, germline and somatic genomic analyses were performed using whole-genome sequencing. The patient’s mutation profile analysis was performed using the ‘somatic n-of-1’ pipeline [7], including assembled reporting based on the ‘Personalised Cancer Genome Reporter’ (PCGR) [8] (Supplementary Materials and Methods). Variants were classified in a number of ways: as pathogenic (P), likely pathogenic (LP), benign, likely benign or variant of unknown significance (VUS) [9]. Currently, there is no quantitative definition of the terms ‘pathogenic’, ‘likely pathogenic’ and ‘variant of uncertain significance’. Guidelines issued by American College of Medical Genetics and Genomics and the Association for Molecular Pathology have been proposed to standardise the variant classification [10]. Therefore, the term ‘pathogenic’ is used where the mutation is disease-causing, ‘likely pathogenic’ means a greater than 90% certainty of a variant being disease-causing, and ‘variant of uncertain significance’ means there is between 10% and 90% certainty that the mutation is disease-causing. These terms are used in order to provide laboratories with a universal, albeit arbitrary, definition. In the future, experimental and statistical approaches will be adopted to objectively assign pathogenetic confidence to variants to more fully inform terminologies and likelihoods [10].

**Figure 2:**
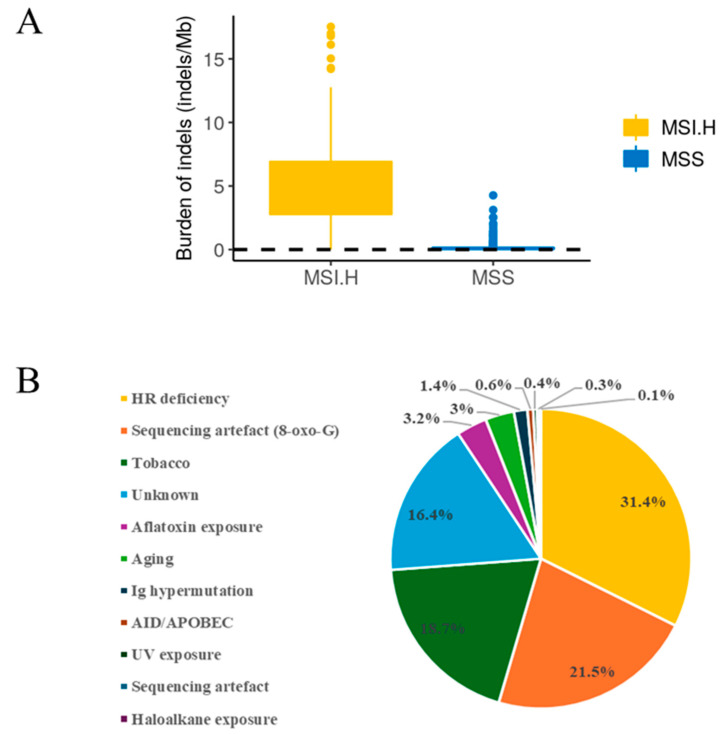
Tumour mutational burden estimate and aetiology of the mutational signature. (**A**) Personalised Cancer Genome Reporter (PCGR) analysis result of the mutational burden of indels (black dashed line) along with the distribution in TCGA samples for samples with known MSI statuses (MSI.H = high microsatellite Instability, MSS = microsatellite stable). The patient was considered to be MSS. (**B**) Pie chart representing the estimate of the relative contribution of known mutational signatures, based on the Mutational Patterns package [11]; Left panel—Signature aetiology types.

Germline DNA analysis did not reveal any abnormalities. However, tumour analysis identified KRAS and TP53 as having LP variants, while SMAD4, PLXNB1, ZIC1, DMBT1, THBS1, NDRG4, CDH13, PTPN1, KDM5C and TTN contained VUS (Table 1). The tumour mutation burden (TMB) was 9.68 mutations/megabase (Figure 2A), in concordance with histological findings of intact mismatch-repair proteins (equivalent to MSS). The mutational signature analysis revealed that three principal risk factors for developing the observed tumour mutational profile were homologous recombination deficiency, sequencing artefact and tobacco exposure (Figure 2B). Closer inspection of the sCNA (somatic Copy Number Variation) analysis revealed several focal and broad gains and losses affecting chromosomes 4, 6, 8, 15, 16, 18 and 21 (Supplementary Table S2). Importantly, this patient’s tumour was found to have a 24-megabase homozygous deletion on chromosome 8 (8p12–8p22; Log ratio = −1.446), affecting 13 genes with a known association to tumour suppression, including TUSC3, SOX7 and WRN genes (Supplementary Table S3).

**Figure 3:**
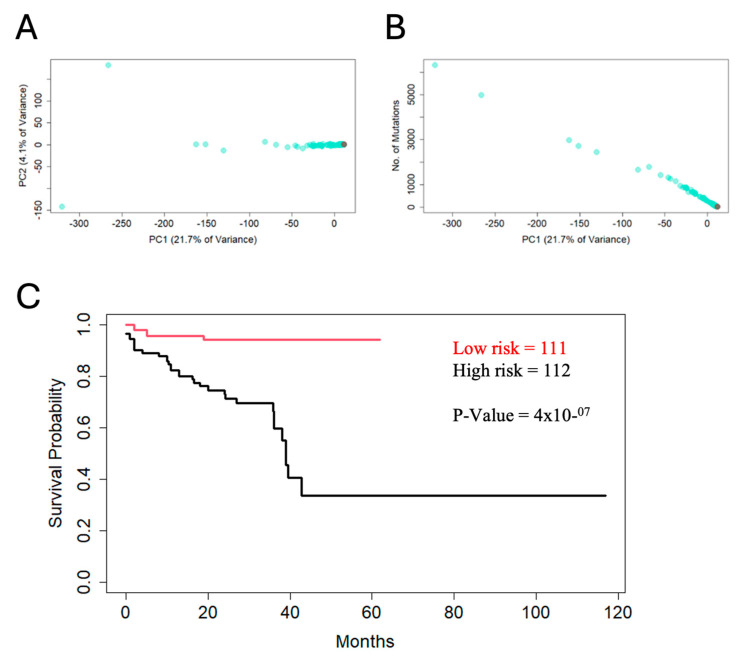
Principal component analysis (PCA) and Overall Survival Kaplan–Meier Curve. PCA plot of the nonsynonymous mutations data in the TCGA COADREAD dataset [13]. (**A**) The red circle represents the patient projected onto the PC space. (**B**) PC1 vs. number of mutations, showing that the variation in the dataset is mostly attributed to the mutational burden. (**C**) The red line represents the low-risk (111 patients), and the black line represents the high-risk patients (112 patients) in the COADREAD TCGA cohort [13]. Cut-off mode was set as median risk from Cox regression.

To further evaluate our findings, we have compared the mutations seen in this patient’s tumour to the colorectal adenocarcinoma reference dataset TCGA COADREAD [12], consisting of 223 patients. There were 8 patients in the COADREAD dataset who had the same mutation as this patient in the TP53 R248W gene, and 31 patients had a KRAS G12D mutation. Interestingly, these mutations were mutually exclusive in the COADREAD cohort. The patients that had the same loss-of-function missense p53 mutation had an average age of 72 years and an average of only 76.87 non-synonymous mutations, close to the median number of mutations of the reference dataset of 78. On the other hand, the patients with the same KRAS protein change had a higher number of mutations, with an average of 186 (higher than 89% of the patients) and an average age of 71 years. Out of the 12 mutated genes classified as LP and VUS (Table 1; Supplementary Table S1), four genes are among the most frequently mutated genes in colorectal cancer. Out of the 223 patients in the COADREAD cohort, the average overlap of nonsynonymous mutations with our patient was 1.6. However, a comparison of overlap might not be enough to understand how this patient fits in within the distribution of the dataset. For this reason, we performed a principal component analysis (PCA) (Figure 3A,B). 

In the COADREAD dataset, most of the variance of the non-synonymous mutations was attributed to the number of mutations (Figure 3A). The first PCA was strongly correlated with mutation burden (Figure 3B), and it explained over 20% of the variance. The tumour in this report fits in with the patients with a significantly lower mutation burden. We then performed a multivariate Cox regression analysis using clinical and mutation variables as predictors of overall survival. The survival analysis revealed a number of mutations and clinical factors to be prognostically relevant, including tumour size, age of the patient and KRAS, TP53, ZIC1, KDM5C, THBS1, CDH13, DMTB1 and NDRG4 gene mutations (Supplementary Table S1). We assessed the overall survival of patients from the TCGA COADREAD dataset stratified with the Cox multivariate model (Figure 3C). The patient was classified as having low risk of cancer-related mortality by the model, i.e., lower than 98% of the patients in the COADREAD cohort.

## 3. Discussion

Individuals without a hereditary syndrome, but with a family history of CRC, are known to be at increased risk for the disease [14]. This risk is considered to be relevant in those who have a first-degree relative (FDR) diagnosed below the age of 50 and in cases with two or more FDRs with CRC. The USA National Cancer Control Network recommends that CRC screening for these patients should begin at either 40 years of age or 10 years before the FDR was diagnosed with colorectal cancer, whichever is earlier [15]. Unfortunately, even with the most stringent of detection policies currently in practice, the patient in this report would not have met the criteria for screening. 

Routine surveillance of this patient after primary surgery and adjuvant chemotherapy revealed liver lesions that were concerning for metastasis. After extensive investigation, these lesions were diagnosed as FNH. FNH is a benign condition that affects healthy liver tissue. Histologically, FNH contains all the cell types seen in normal hepatic tissue, but their arrangement is abnormal [16]. FNH does not cause any symptoms, so they are generally diagnosed incidentally during routine abdominal imaging. They have a distinctive appearance radiologically, allowing the diagnosis to be made [17]. However, FNH can occur post-systemic chemotherapy, leading to concern for recurrence of the disease. The exact pathogenesis of FNH is poorly understood, but in the case of patients who have received oxaliplatin, it is thought that regeneration of damaged liver parenchyma post-chemotherapy leads to FNH [18]. 

The unusually young age of the patient and family history has prompted a molecular profiling focused on identifying potential oncogene driver mutations and chromosomal aberrations. While the germline mutation analysis did not reveal any known cancer predisposition gene mutations, the tumour DNA analysis of this patient revealed a number of missense mutated cancer-associated genes (Table 1) and a loss of several chromosomal regions (Supplementary Table S2). The identified TP53 mutation is known to increase the lifetime risk of CRC by 2.8 times [19], while KRAS G12D mutations are less commonly seen in EOCRC and are generally associated with later-onset CRC and a worse prognosis [20,21]. Approximately 5.0–24.2% of colorectal cancers (CRCs) have inactivating mutations in SMAD4, and this is associated with poor prognosis and the aggressive clinicopathological characteristics of CRC [22]. TTN is one of the top ten most commonly mutated genes in CRC, and in some populations, TTN mutations confer a positive response to immunotherapy [23]. The Cox multivariate regression analysis provided further information regarding a favourable prognosis for this patient. The tumour mutation burden (TMB) was 9.68 mutations/megabase, which is considered below the threshold of a high TMB (10 or more mutations/megabase) [24]. With a low TMB and microsatellite stability observed, the patient would be unlikely to respond to immunotherapy.

Among the numerous genomic imbalances in CRC, the loss of 8p chromosome has been reported in MSS CRC cases [25,26]. This patient was found to have a 24-megabase deletion on chromosome 8 affecting a number of cancer-associated genes. The absence of some of these genes such as TUSC3 is associated with a favourable prognosis [27]. The deletion of some genes like SOX7, which has a tumour suppressor role, is associated with a worse prognosis [28]. Interestingly, homozygous deletion has affected a member of the RECQ helicase family of genes: the Werner syndrome (WRN) gene. Due to the critical role of RECQ genes in DNA repair and genomic stability maintenance, RECQ helicases are associated with CRC pathogenesis [29]. The deletion of the WRN gene is commonly associated with a high level of genomic instability and is often implicated in MSI cancers [30], contrary to the patient in this study presenting with an MSS tumour. However, low expression of WRN in CRC renders these malignancies susceptible to specific DNA-damaging chemotherapeutic agents, including 5-fluorouracil and oxaliplatin [31,32], both of which this patient received as part of the chemotherapeutic regimen. Of note, DNA-damaging chemotherapeutic agents can also increase somatic mutational burden as reported by Christensen and Pich [33,34]. Namely, the authors demonstrate that mutation rate in surviving cancer cells is more than 50-fold higher than in the normal mutational aging process. However, the related treatment resistance mechanism in some cases can also be attributed to clonal expansions. The possibility that the positive response to chemotherapy in this patient is associated with deletions of other genes on chromosome 8p remains unclear.

## 4. Conclusions

Despite a number of adverse clinical features, such as the very young age at diagnosis and acute presentation with intestinal perforation, this patient has had a favourable clinical course thus far. From the TCGA cohort, we observed that this patient’s tumour profile was more commonly seen in patients older than seventy years of age; this coupled with the absence of WRN may confer a better prognosis for this patient. This report highlights the importance and the potential of molecular profiling to assess clinical interventions and to inform the EOCRC post-treatment surveillance. 

## Figures and Tables

**Table 1:** Whole-genome sequencing (NGS) Personalised Cancer Genome Reporter (PCGR) results from the tumour.

Gene	Exon	Protein Change	Nucleic Acid Mutation	Variant Class
KRAS	exon 2	p.Gly12Asp	c.35G>A	LP Variants of Likely Pathogenic Significance
TP53	exon 7	p.Arg248Trp	c.742C>T	
SMAD4	exon 10	p.Gly419Trp	c.1255G>T	VUS Variants of Uncertain Clinical Significance
PLXNB1	exon 22	p.Gly1423Cys	c.4267G>T	
ZIC1	exon 1	p.Thr297Met	c.890C>T	
DMBT1	exon 38	p.Cys1552Ser	c.4654T>A	
THBS1	exon 9	p.Gly454Cys	c.1360G>T	
NDRG4	exon 16	p.Ala370Val	c.1109C>T	
CDH13	exon 8	p.Gly353Ter	c.1057G>T	
PTPN1	exon 6	p.Arg221Met	c.662G>T	
KDM5C	exon 10	p.Gly452Cys	c.1354G>T	
TTN	exon 326	p.Leu28470Val	c.85408C>G	

## Data Availability

The data are in the process of submission to the European Genome-Phenome Archive (EGA), a protected online repository for hosting clinical data.

## Supplementary Materials

The following supporting information can be downloaded at: https://www.mdpi.com/article/10.3390/jpm14060639/s1, Table S1. Multivariate Cox Regression for Overall Survival based on clinical and pathological features. SE—standard error. Significant *p*-values highlighted in bold. Table S2. Personalised Cancer Genome Reporter (PCGR) chromosomal instability analysis results for the tumour. Chromosomal segment—chromosome number followed by start and end position; segment length megabase (MB) value; Log Ratio—positive and negative value, for segment gain and loss, respectively; event type—broad or focal. Table S3: sCNA (somatic Copy Number Variation) analysis results for cancer-associated genes on chromosome 8. Gene symbol; Gene Name; Chromosomal segment—chromosome number, start and end position.
