# Supplementary material for: Genomic and Transcriptomic Analysis of a Patient with Early-Onset Colorectal Cancer and Therapy-Induced Focal Nodular Hyperplasia: A Case Report
Source: J Pers Med. 2024 Jun 15;14(6):639. doi: 10.3390/jpm14060639 (PMC11204579; doi:10.3390/jpm14060639)
Supplement: Supplementary file 1 [file jpm-14-00639-s001.zip › jpm-3016514-supplementary.pdf]

**Supplemental data**

Supplemental Table S1. Multivariate Cox Regression for Overall Survival based on clinical and pathological features. SE - standard error. Significant P values highlighted in bold.

| Variable | Coefficients | SE (Coefficient) | P-value |
|---|---|---|---|
| **Lymph node Stage: N2** | **1.566168** | **0.330852** | **2.20E-06** |
| Lymph node Stage: N1b | -20.8274 | 43635.87 | 0.999619 |
| Lymph node Stage: N1 | -0.12928 | 0.451136 | 0.774446 |
| Lymph node Stage: N2a | -16.4087 | 45044.82 | 0.999709 |
| Rectum | -0.73874 | 0.420725 | 0.07911 |
| Cecum | -0.70427 | 0.383579 | 0.06635 |
| Ascending Colon | -0.37758 | 0.457874 | 0.409577 |
| Descending Colon | -0.09332 | 0.731384 | 0.89847 |
| Transverse Colon | 0.413982 | 0.604313 | 0.493315 |
| Sigmoid Colon | -1.20682 | 0.445007 | 0.00669 |
| Hepatic Flexure | 0.527133 | 0.733024 | 0.472066 |
| Splenic Flexure | -0.41201 | 1.023062 | 0.687154 |
| Rectosigmoid Junction | 1.778123 | 0.74561 | 0.017089 |
| AJCC Tumour Stage Code: T3 | 0.226892 | 0.381172 | 0.551678 |
| AJCC Tumour Stage Code: T2 | -0.61901 | 0.728314 | 0.395372 |
| **AJCC Tumour Stage Code: T4** | **1.606711** | **0.447427** | **0.000329** |
| **Age** | **0.046981** | **0.013578** | **0.00054** |
| Gender: Female | 0.368668 | 0.323521 | 0.254474 |
| **No. Mutations** | **-0.00183** | **0.000274** | **2.32E-11** |
| **KRAS** | **-0.71053** | **0.337987** | **0.035533** |
| **TP53** | **-0.94261** | **0.324816** | **0.003708** |
| TTN | 0.6454 | 0.331183 | 0.051323 |
| **ZIC1** | **8.316058** | **1.025217** | **5.00E-16** |
| KDM5C | -1.63656 | 0.741345 | 0.027276 |
| SMAD4 | 0.645602 | 0.45269 | 0.153826 |
| **THBS1** | **1.468005** | **0.530649** | **0.005667** |
| PLXNB1 | -15.0342 | 4917.707 | 0.997561 |
| **CDH13** | **3.190195** | **0.75598** | **2.44E-05** |
| **DMBT1** | **-4.73145** | **1.02518** | **3.93E-06** |
| **NDRG4** | **4.488365** | **1.143605** | **8.68E-05** |
| PTPN1 | -14.6279 | 2955.821 | 0.996051 |

Supplemental Table S2. Personalised Cancer Genome Reporter' (PCGR) chromosomal instability analysis results for the tumour. Chromosomal segment – chromosome number followed by start and end position; Segment length megabase (MB) value; Log Ratio – positive and negative value, for segment gain and loss, respectively; event type – broad or focal.

| Chromosomal segment | Segment length (MB) | Log R | Event type |
|---|---|---|---|
| chr4:153276999-189549276 | 36.272 | -0.802 | broad |
| chr8:12613531-37145961 | 24.532 | -1.446 | broad |
| chr8:51307370-75313553 | 24.006 | 0.84 | focal |
| chr18:51208501-69150727 | 17.942 | -0.807 | broad |
| chr18:384153-15042098 | 14.658 | -0.804 | broad |
| chr18:69294340-78016309 | 8.722 | -0.805 | focal |
| chr8:164860-71548 | 6.967 | -1.456 | focal |
| chr18:38180664-44471541 | 6.291 | -0.864 | focal |
| chr8:139420362-145027855 | 5.607 | 0.822 | focal |
| chr8:42601586-46850220 | 4.249 | 0.9 | broad |
| chr8:8232443-12148644 | 3.916 | -1.415 | focal |
| chr18:27137107-30241431 | 3.104 | -0.847 | focal |
| chr8:46850531-48980850 | 2.130 | 0.864 | focal |
| chr21:14339867-15896600 | 1.557 | -1.438 | focal |
| chr16:21392689-22699303 | 1.307 | 0.971 | focal |
| chr21:15996236-17085531 | 1.089 | -1.469 | focal |
| chr8:7138465-8204572 | 1.066 | -1.224 | focal |
| chr8:41884175-42599971 | 0.716 | 1.49 | focal |
| chr16:18099913-18796468 | 0.697 | 0.845 | focal |
| chr8:38606727-39284877 | 0.678 | -1.448 | focal |
| chr8:12148928-12608523 | 0.460 | -1.007 | focal |
| chr15:101753918-101980585 | 0.227 | 0.875 | focal |
| chr8:41735999-41883748 | 0.148 | 1.874 | focal |
| chr18:69152402-69294129 | 0.142 | -1.245 | focal |
| chr6:76182448-76322000 | 0.140 | 0.989 | focal |
| chr8:8208060-8229113 | 0.021 | -2.356 | focal |
| chr6:58449983-58453090 | 0.003 | 0.963 | focal |

Supplemental Table S3: sCNA (somatic Copy Number Variation) analysis results for cancer associated genes on chromosome 8. Gene symbol; Gene Name; Chromosomal segment – chromosome number, start and end position.

| **SYMBOL** | **GENE NAME** | **SEGMENT** |
|---|---|---|
| WRN | WRN RecQ like helicase | chr8:12613531-37145961 |
| UNC5D | unc-5 netrin receptor D | chr8:12613531-37145961 |
| TUSC3 | tumour suppressor candidate 3 | chr8:12613531-37145961 |
| SOX7 | SRY-box transcription factor 7 | chr8:8232443-12148644 |
| RHOBTB2 | Rho related BTB domain containing 2 | chr8:12613531-37145961 |
| ARHGEF10 | Rho guanine nucleotide exchange factor 10 | chr8:164860-7131548 |
| NKX3-1 | NK3 homeobox 1 | chr8:12613531-37145961 |
| MTUS1 | microtubule associated scaffold protein 1 | chr8:12613531-37145961 |
| MCPH1 | microcephalin 1 | chr8:164860-7131548 |
| LZTS1 | leucine zipper tumour suppressor 1 | chr8:12613531-37145961 |
| DUSP4 | dual specificity phosphatase 4 | chr8:12613531-37145961 |
| DLC1 | DLC1 Rho GTPase activating protein | chr8:12613531-37145961 |
| CLU | clusterin | chr8:12613531-37145961 |
